# Standardised Chinese herbal treatment delivered by GPs compared with individualised treatment administered by practitioners of Chinese herbal medicine for women with recurrent urinary tract infections (RUTI): study protocol for a randomised controlled trial

**DOI:** 10.1186/s13063-016-1471-5

**Published:** 2016-07-27

**Authors:** Andrew Flower, Kim Harman, George Lewith, Michael Moore, Felicity L. Bishop, Beth Stuart, Nicholas Lampert

**Affiliations:** 1 Health Research, Complementary and Integrated Medicine Research, University of Southampton, Aldermoor Close, Southampton, SO16 5ST UK; 2 Primary Care & Population Science, University of Southampton, Southampton, UK; 3 Health Research, Complementary and Integrated Medicine Research, University of Southampton, Southhampton, UK; 4 Psychology, University of Southampton, Faculty of Social, Human and Mathematical Sciences, Southampton, UK; 5 Birmingham Centre for Chinese Medicine, Birmingham, B14 6DT UK

**Keywords:** Urinary tract infection, Chinese herbal medicine, Primary care, feasibility

## Abstract

**Background:**

In the UK, urinary tract infections (UTIs) are the most common bacterial infection presented by women in primary care. Recurrent urinary tract infections (RUTIs) are defined as three episodes of UTI in the last 12 months, or two episodes in the last 6 months. Between 20 and 30 % of women who have had one episode of UTI will have an RUTI, and approximately 25 % of these will develop subsequent recurrent episodes. RUTIs can have a significant negative effect on the quality of life, and have a high impact on health care costs as a result of outpatient visits, diagnostic tests and prescriptions. Chinese herbal medicine (CHM) has a recorded history of treatments for the symptoms of UTIs for more than 2000 years. More recent clinical research in China has provided some preliminary evidence that CHM can alleviate the symptoms of UTIs and reduce the rate of recurrence, but more rigorous investigation is required.

**Methods/design:**

The RUTI trial is a double-blind, randomised, placebo-controlled, feasibility trial. A total of 80 women will be randomised to ‘individualised’ herbs prescribed by a Chinese herbal practitioner or to ‘standardised’ herbs provided by primary care clinicians. Both arms will have herbs for prevention of UTIs and treatment of acute episodes. Treatment duration is for 16 weeks.

The primary outcomes are the number of episodes of recurrent UTIs during the trial period and in the 6 months of follow-up, and the number of days of symptoms rated moderately bad or worse based on patient diaries. Secondary outcomes will assess participant expectations and beliefs, adherence to the treatment, adverse events and health economics and provide quantitative and qualitative assessments of the impact of recurrent infections on the lives of women.

**Discussion:**

The RUTI trial is the first instance of CHM delivered as a clinical trial of an investigatory medicinal product in the UK. This study provides important information regarding the feasibility and acceptability of researching and using CHM in Primary care. Once completed, it will provide provisional estimates of the variance of change in continuous outcomes to inform a power calculation for a larger, more definitive trial.

**Trial registration:**

EudraCT, 2013-004657-24. Registered on 5 September 2014.

**Electronic supplementary material:**

The online version of this article (doi:10.1186/s13063-016-1471-5) contains supplementary material, which is available to authorized users.

## Background

### Epidemiology and current management

In the UK, urinary tract infections (UTIs) are the most common bacterial infection presented by women in the primary care setting [1, 2], with approximately 40–50 % of women experiencing one episode during their lives [3]. This accounts for between 1–3 % of all consultations in general practice [4].

Recurrent urinary tract infections (RUTIs) are commonly defined in the literature as three episodes of UTI in the last 12 months or two episodes in the last 6 months [5]. Between 20 and 30 % of women who have had one episode of UTI will have a recurrent UTI and approximately 25 % of these will develop subsequent recurrent episodes [6, 7]. RUTIs can have a significant negative effect on the quality of life [8] and have a high impact on healthcare costs as a result of outpatient visits, diagnostic tests and prescriptions.

Antibiotics are currently the mainstay treatment for both acute and recurrent UTIs. Whilst antibiotics may be effective in reducing the duration of severe symptoms in acute episodes [9, 10], antibiotic resistance is currently estimated at 20 % for trimethoprim and cephalosporins and 50 % for amoxicillin [11]. Antibiotic resistance and previous episodes of cystitis have been positively associated with an increased duration of severe symptoms of UTIs [2]. It is predicted that antibiotic resistance will continue to increase [12]. The UK government and Chief Medical Officer for England have indicated the need to consider alternatives to antibiotics [13].

Antibiotic prophylaxis is, however, used to prevent RUTIs. Treatment usually lasts for between 6 and 12 months but can be extended for up to 5 years [14]. A Cochrane systematic review of antibiotics used to prevent RUTIs in non-pregnant women found that antibiotics given continuously for 6–12 months were significantly more able than placebo to prevent recurrent infection (RR 0.15 (95 % CI 0.08 to 0.28 and NNT 1.85 (CI 1.60 to 2.20)) [5]. However, more severe side effects were also experienced, requiring the withdrawal of treatment (such as urticaria, nausea and vomiting) and less serious but unpleasant side effects such as oral and vaginal candidiasis, and gastrointestinal disturbances resulting from antibiotic treatment. These can cause considerable discomfort and probably contribute to women’s expressed preference to avoid antibiotics use [15].

Once prophylaxis is discontinued, even after extended periods, approximately 50–60 % of women will become re-infected within 3 months [16, 17]. Thus, antibiotic prophylaxis does not exert a long-term effect on the baseline infection rate.

### Rationale for use of Chinese herbal medicine

Chinese herbal medicine (CHM) has a recorded history of treating the symptoms of UTIs for over 2000 years [18]. More recent clinical research in China has provided some supportive evidence that CHM can alleviate the symptoms of UTIs [19–22] and reduce the rate of recurrence 1 year post-treatment from 30 % when antibiotics were used alone to 4.4 % when antibiotics and CHM were combined [20]. A recent Cochrane review of CHM for RUTIs found some preliminary supportive data for herbal interventions, but the trials were of poor quality and further and more rigorous research was recommended [23].

### Rationale for the trial design

In accordance with MRC recommendations for the investigation of poorly evidenced complex interventions such as CHM [24], a strategic, phased, research-development process is essential. The RUTI trial has been conceived as a small scale feasibility trial to explore the treatment effects of CHM and to investigate if CHM can be used and researched in primary care.

### Research objectives

The key objectives for this study are as follows:

- To explore the feasibility of developing, implementing and evaluating a CHM intervention within the primary care networks with particular regard to recruitment, referral patterns, patient compliance, the use of a plausible herbal placebo and dropout rates
- To evaluate the acceptability and sensitivity to change of different outcomes measures and to provide a provisional estimation of any effect size relating to CHM treatment that could inform a more definitive future study
- To use nested qualitative research and quantitative quality of life outcome measures to explore the experience of women taking CHM and to investigate any perceived differences between the delivery of standardised remedies via GP practice nurses and individualised remedies via CHM practitioners

## Methods/design

The trial has two separate but linked arms (see Additional file 1). Forty women from the Wessex, South and Northwest London and the Brighton and Hove areas of the Clinical Research Network (CRN) will be randomised to take traditional individualised herbal prescriptions following consultation with a registered CHM practitioner at a private clinic. Forty women from Wessex will be randomised to standardised ‘prescriptions’ of herbal capsules administered via nurses and doctors from GP practices. Both arms will have equal allocation to active and placebo medications. The trial is fully blinded with only the herbal dispensaries knowing the treatment allocation of the women.

### Eligibility

A medical record search will be conducted for women who have had more than two prescriptions for trimethoprin or nitrofurantoin within the last 12 months and/or have been recorded as having recurrent UTIs.

A standard letter will be sent to these women with the Patient Information Sheet explaining the trial (Additional file 2). They will be contacted by the trial manager who will explain the trial more fully and assess eligibility. If the women are both eligible and interested an appointment will be made at the primary care site or at the appropriate clinic where written consent will be taken (Additional file 3).

### Inclusion/exclusion criteria

Women will be eligible for the trial if they meet all of the following criteria:

- are over 18 years and under 65 years of age
- have reported three or more uncomplicated recurrent lower UTIs in the previous 12 months
- have had at least one episode documented as being suspected or confirmed as a bacterial UTI by a GP

Women will be ineligible if they have any of the following criteria:

- have symptoms of complicated UTIs, such as acute pyelonephritis.
- have known hepatic or renal disease
- are pregnant or breast feeding
- have diabetes
- are taking drugs which may interact with Chinese herbal medicine: cardiac glycosides (digoxin), warfarin and lithium
- have psychosis, dementia or terminal illness that may prevent completion of the symptom diaries
- have commenced a new treatment (conventional or complementary or alternative medicine (CAM)) for RUTIs in the previous 6 months.

### Randomisation and blinding

Computer-generated block randomisation to either verum (active) or placebo CHM treatment will be performed with random block sizes of two, four, or six participants to ensure equal numbers in groups and to prevent the study team and the dispensary team from being able to predict a pattern to the randomisation.

In the standardised arm, this randomisation sequence will be used by the manufacturer to code the herbal capsules prior to the capsules being sent to GP practices. Capsules will be dispensed according to this sequence, and a record of the batch number recorded by the practice nurse.

For the individualised arm, the randomisation sequence will be stored at the herbal dispensary in opaque sealed envelopes and kept in a safe place only accessible to a designated person. On receiving the formula by email, this person will open the envelope and inform the herbal dispensary whether the trial participant is to receive the placebo or active herbs. A record of this allocation will be kept by the designated person so that repeat prescriptions will be allocated to the same arm.

### Intervention

All women taking part will be able to consult with their GPs and, if necessary, use conventional medicines such as antibiotics for acute infections. Antibiotic use will be recorded as one of the trial outcomes. These can be taken in conjunction with the ‘acute’ herbal treatment.

Participants in the standardised arm will receive ‘acute’ and ‘preventative’ herbal capsules (Size 0, hypromellose capsules) to be used during and between instances of infection. Matching placebo capsules have been prepared containing therapeutically inert sugar beet fibre. In total, 16 weeks of preventative herbal capsules will be allocated to each participant, with an additional 4 weeks of ‘acute’ capsules to be used in the instance of a UTI occurring.

The individualised arm of the trial will follow routine practice of CHM, which involves a practitioner writing an individualised herbal prescription typically comprising between ten and 15 herbs selected from the Chinese herbal material medica. The selection of herbs is contingent upon a full CHM differential diagnosis made after a detailed consultation process. The formula used will also change over time to address the changing clinical presentation of the patient.

After the consultation with the CHM practitioner verum or placebo herbal medicines will be dispensed as concentrated herbal granules to be taken twice a day. Each dose will be separately packaged in a sealed foil sachet. These granules dissolve in hot water for patients to drink as an herbal decoction. The practitioner may allocate preventative treatment with a clearly labelled standby for an acute infection. Women allocated to placebo treatment will receive identically packaged granules derived from therapeutically inert food colourings and flavourings. Once re-constituted, these placebo herbs resemble the colour and the strong taste and smell of the verum formulae.

### Standardised trial medicines

Chinese herbal medicine in the UK involves the use of plant-based herbal medicines. The standardised herbal formulae have been developed through expert consultation and a literature review conducted prior to the trial. Each formula consists of three herbs (see Additional file 4).

These two formulae are comprised of the following herbs:

$$ \begin{array}{l}\mathrm{RUTI}\hbox{-} \mathrm{a}:\\ {}\mathrm{B}\mathrm{a}\mathrm{i}\ \mathrm{H}\mathrm{u}\mathrm{a}\ \mathrm{She}\ \mathrm{She}\ \mathrm{C}\mathrm{a}\mathrm{o}\ \left(\mathrm{Herba}\ \mathrm{H}\mathrm{edyotis}\ \mathrm{Diffusa}\right)\ \left(40\%\ \mathrm{o}\mathrm{f}\ \mathrm{the}\ \mathrm{Formulation}\right)\\ {}\mathrm{H}\mathrm{u}\mathrm{a}\mathrm{n}\mathrm{g}\ \mathrm{B}\mathrm{a}\mathrm{i}\ \left(\mathrm{Cortex}\ \mathrm{Phellodendron}\ \mathrm{Amurense}\right)\ \left(20\%\ \mathrm{o}\mathrm{f}\ \mathrm{the}\ \mathrm{Formulation}\right)\\ {}\mathrm{J}\mathrm{i}\mathrm{n}\ \mathrm{Qian}\ \mathrm{C}\mathrm{a}\mathrm{o}\ \left(\mathrm{Hebra}\ \mathrm{Lysimachia}\ \mathrm{C}\mathrm{hristinae}\right)\ \left(40\%\ \mathrm{o}\mathrm{f}\ \mathrm{the}\ \mathrm{Formulation}\right)\end{array} $$

$$ \begin{array}{l}\mathrm{RUTI}\hbox{-} \mathrm{p}:\\ {}\mathrm{Huang}\ \mathrm{Qi}\ \left(\mathrm{Radix}\ \mathrm{Astragalus}\ \mathrm{Membranaceus}\right)\ \left(50\%\ \mathrm{o}\mathrm{f}\ \mathrm{the}\ \mathrm{Formulation}\right)\\ {}\mathrm{W}\mathrm{u}\ \mathrm{Y}\mathrm{a}\mathrm{o}\ \left(\mathrm{Radix}\ \mathrm{Lindera}\ \mathrm{Strychnifolia}\right)\ \left(25\%\ \mathrm{o}\mathrm{f}\ \mathrm{the}\ \mathrm{Formulation}\right)\\ {}\mathrm{K}\mathrm{u}\ \mathrm{Shen}\ \left(\mathrm{Radix}\ \mathrm{Sophorae}\ \mathrm{Flavescentis}\right)\ \left(25\%\ \mathrm{o}\mathrm{f}\ \mathrm{the}\ \mathrm{Formulation}\right)\end{array} $$

In terms of modern pharmacology, the herbal formulae are reported to have the following main functions (see Additional file 4):

- RUTI-a has antibacterial and anti-inflammatory actions.
- RUTI-p has immune enhancing, anti-inflammatory, antibacterial, and mildly diuretic actions.

These standardised herbs will be administered as 0.4 g capsules (two pills taken twice a day (b.d.) for preventative treatment, and four pills taken four times a day (q.d.) in the event of an acute infection).

### Concealment and emergency unblinding

Concealment will be maintained with packaging in the standardised arm that does not provide any information as to the contents and in the individual arm by the use of identical packaging and by the participant’s herbs not being seen by the CHM practitioner.

Each woman enrolled will be provided with an emergency unblinding card with information on how to access the trial team if required.

Emergency unblinding will be possible by a member of the study team not conducting study assessments, who will use the participant ID number and check allocation by accessing the centralised allocation list stored at the herbal dispensary. Unblinding will be required in case of the following:

- A compelling medical need as assessed by the treating medical doctor, e.g. a serious adverse event (SAE) has occurred, and the treatment allocation is required to inform subsequent clinical care
- Ingestion of study products by a child
- Ingestion of excessive amounts of study products either by the participant or by an adult not participating in this study.

### Outcomes (see Figs. 1 & 2)

#### Primary outcome measures

The primary outcomes will be the number episodes of recurrent UTIs during the trial period and in the 6 months of follow up and the number of days of symptoms rated moderately bad or worse according to the patient diaries [10]. These will be assessed by symptom diaries, which will indicate the severity of acute and recurrent UTI symptoms (reporting dysuria, haematuria, frequency during day and night, ‘smelly urine’, ‘tummy pain’, generally feeling unwell, and restriction of daily activities). These diaries have been used in this format for acute respiratory and urinary tract infections and have been validated and shown to be sensitive to change [2, 10].

**Fig. 1:**
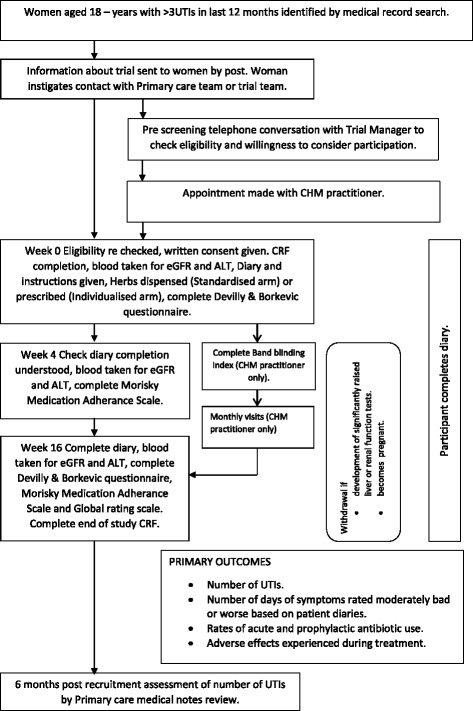
RUTI flow diagram

**Fig. 2:**
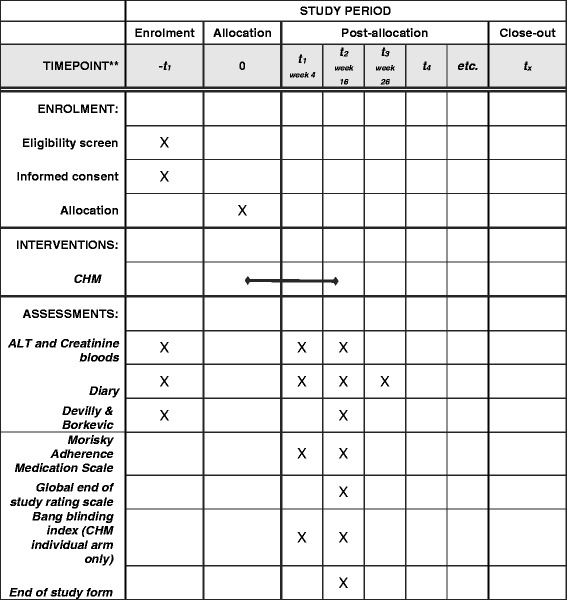
SPIRIT: schedule of enrolment, interventions, and assessments

In addition, participants will also be asked to complete an end-of-month diary in which they record the number of days with symptoms, changes in presentation, time lost from work, and how easy it has been to comply with the herbal medicine regime. These diaries will provide information on the rate and severity of acute infection but also on the impact of low-grade, chronic infection on the lives of women. Participants will be asked to complete both an acute and a monthly diary for the 6-month follow-up to the trial to enable the evaluation of any longer-term changes in infection rates.

#### Secondary outcomes include the following:

- The use of antibiotics for acute UTIs elicited from symptom diaries and cross-referenced with GP notes
- The Credibility/Expectancy questionnaire, which is used to assess participant attitudes on the effectiveness of CHM prior to and on completion of the trial [25]
- The Morisky Medication Adherence Scale, which is used to assess compliance with the herbal medicines. In addition participants will be asked to return all herbal containers including those that are empty and those with any unused herbal capsules or granules. These will be counted to help assess whether herbal medicines were taken as instructed.
- At the end of trial, a Global Ratings of Change pro forma will be used to assess participant perceptions of the overall change in the frequency and severity of their urinary symptoms. An 11-point Likert scale will be used to assess these changes and an improvement of two or more points will be considered as a clinically important change [26].
- Liver (ALT) and renal (serum creatinine) function tests will be conducted at weeks 0, 4 and 16. Blood results will be used to assess safety of the herbal preparations to ensure that no change in kidney or liver functions occurs (each site was given the specific normal reference ranges for their associated NHS laboratory where the blood was processed.)
- Economic and quality of life assessment using the validated EQ5D outcome measure.
- An assessment of blinding using the Bang blinding index (CHM practitioner only).
- Two nested qualitative studies will also be conducted during this trial. On completion of the trial, semi-structured phenomenological interviews will be conducted with purposively sampled women from all four groups to provide an in-depth exploration of their subjective experiences of taking CHM and conventional care during the trial. We will also investigate the enablers and barriers that affected women’s decisions to participate in the trial. A second study will include interviews of GP practice nurses to explore their experiences of delivering CHM in a clinical trial conducted in primary care.

### Research ethics and governance

Obtaining regulatory approval from the Research Ethics Committee, the MHRA, and NHS Research and Development has been an important outcome for this trial. Initial submissions were made to the MHRA in October 2014, and final approval was granted in November 2015. Requests for ethical approval were made in August 2014 and multi-centre approval was granted by the London Surrey Borders Research Ethics Committee in December 2015. (Ref: 14/LO/1425 and by the MHRA (EudraCT number: 2013-004657-24). The lead research and development organisation was the Wessex Clinical Research Network. A more detailed account of the unique challenges required to gain these approvals has been provided in the discussion section. In addition the following ethical issues required clarification:

All women participating in the trial will be able to continue their pattern of usual care alongside taking CHM (see Additional files 2 and 3). Antibiotics can be accessed from their GPs as a rescue package in the event of a serious UTI occurring.

All herbs to be used in the individualised arm of the trial are currently in daily usage within the UK under Regulation 3 of The Human Medicines Regulations 2012 (formally Section 12(1) of the Medicines Act 1968), which permits unlicensed herbal remedies to be made up and supplied by a practitioner to meet the needs of an individual patient following a one-to-one consultation. Regulation 3 remedies are not subject to a regime of specific safety or quality requirements and currently there are no restrictions in terms of those who operate under this regime.

### Data management

#### Confidentiality

All point-of-care test specimens, evaluation forms, reports and other records will be identified in a manner to maintain participant confidentiality and in accordance with the Data Protection Act 1998. All records will be kept in a secure storage area on site with access restricted only to staff delegated duties on the study. Clinical information will not be released without the written permission of the participant, except in cases of emergency or as necessary for monitoring and auditing by the study sponsor. All study staff must comply with the Data Protection Act 1998 and abide by the requirements of the University of Southampton relating to the collection, storage, processing and disclosure of personal information. Any data from this research will be stored according to the University of Southampton Data Protection policy. Written and digitally recorded data will be stored on a password protected computer that will be kept in a secure environment. Any papers connected with this trial will be kept in secure University storage for 10 years and will only be viewed by the research team.

### Data monitoring committee

An independent data monitoring committee has been established to review the results of any abnormal blood tests, adverse events, and other relevant data to decide whether or not the trial should continue. The committee will comprise of a medical statistician, a consultant urologist and a medical Toxicologist.

### Data analysis

The primary outcomes from this study are descriptive, relating to the feasibility of recruitment, the acceptability of randomisation, study medications and outcome measures including patient diaries, and the retention of randomised patients in the study. We will also describe additional data on adverse effects, antibiotic use and adherence to medication, beliefs and expectations of participants, health economics and changes in quality of life.

Although the trial is not powered to provide estimates of effect, exploratory analysis will be undertaken to estimate any effect of individualised, standardised and placebo treatments on the primary outcomes of reducing the frequency and severity of recurrent UTIs.

Continuous outcomes will be explored using linear regression, provided the assumptions underlying the model are met. If not, non-parametric methods will be used. Binary measures will be analysed using logistic regression and count models using an appropriate count distribution. All analyses will be on an intention-to-treat basis and will control for important confounders. Results will be reported in accordance with CONSORT guidelines.

We will analyse any missing data to see if any recurrent patterns emerge, and we will record the numbers and reasons given for participant withdrawal or loss to follow-up, as these are key feasibility outcomes that will inform a larger trial.

Data from the trial will be entered into SPSS 22 for Windows (SPSS Inc., Chicago) and the analysis completed using Stata 14 (StataCorp).

### Nested qualitative study

All qualitative interviews will be digitally audio-recorded (with permission) and transcribed verbatim with the participant details being anonymised.

The qualitative research investigating the experience of trial participants will not commence until all the quantitative outcomes have been completed. Those conducting the interviews will remain blinded to any quantitative analysis of the participants’ case record forms. An interpretative phenomenological analysis (IPA) [27] will be used to explore the subjective experiences of these women with a particular emphasis on their perceptions of any changes in health and wellbeing during the trial, the meanings attributed to these changes, and the rationales they used to explain them-including their attitudes and beliefs in relation to CHM.

In addition, in order to explore the feasibility of administering CHM in primary care, practice nurses involved in the trial will also be interviewed. An inductive thematic analysis will be undertaken to identify key themes that reflect the shared experiences of nurses involved in the trial [28, 29].

## Discussion

The regulatory status of herbal medicinal products being used within a research context in the UK was far from clear at the outset of this study. After discussion with the Medicines and Healthcare products Regulatory Agency (MHRA), the body responsible for regulating clinical trials in the UK, it became clear that any pre-formulated herbal product designed for use in a clinical trial setting was considered as an Investigational Medicinal Product (IMP) and had to abide by the regulatory requirements of a clinical trial of an IMP (CTIMP). These requirements have been established for testing novel pharmaceuticals and present a significant challenge to herbal medicinal products for a number of reasons.

First, whereas a pharmaceutical usually involves a single, refined, active compound, herbal medicines may contain hundreds or even thousands of compounds of variable consistency that may contribute to any therapeutic effect observed. Second, the production of a herbal medicine involves a complex trail from ‘fields to pharmacy’ that cannot replicate the sterile, controlled facilities of a pharmaceutical company that is bound by stringent, properly audited, and certified Good Manufacturing Practices (GMP). Third, the stability of an herbal product over time is difficult to assess, despite generous shelf lives being ascribed to them by herbal manufacturers.

These challenges were gradually overcome through on-going dialogue with the MHRA. Eventually it was agreed that key marker compounds could be used to authenticate an herb used in a trial, and that these compounds should also be used to establish the stability of the finished herbal product. Second, it is possible to locate herbal manufacturers in China and Taiwan who have either EU or Australian certification for GMP. This included Good Agricultural Practice (GAP), as well as guaranteeing quality assured processes of production for the herbal granules to be used in the trial. These ensured that the herbs were properly evaluated with regard to authentication, bio-burden, and contamination with heavy metals or pharmaceuticals, and that the finished product met pre-agreed standards.

However, as this feasibility study was considered a CTIMP, any products being tested were required to be manufactured by a company with MHRA Manufacturers Authorisation for CTIMPs, which would be able to provide the extensive and detailed information required for the Investigators Brochure that provides a key part of the application for a trial license. Fortunately, we were able to collaborate with Essential Nutrition Ltd., a company with a CTIMP Manufacturers Authorisation and experience of producing herbal and nutritional supplements and a Qualified Person who could take legal responsibility for signing out the finished product to be used in the clinical trial. We will discuss the intricacies, complexities, and fairly substantial costs involved in satisfying these regulatory processes in more details in a separate paper.

Whilst pre-formulated herbal products were considered as CTIMPs, individualised herbal prescriptions were instead considered as being routine practice of herbal medicine and were not compelled to go through the same stringent regulatory processes. This is an important precedent for future research on herbal medicines.

As the trial aims to recruit via NHS pathways, regulatory approval was also needed from an NHS Research Ethics Committee (REC) and an NHS Research and Development (R & D) committee. In addition, ethical approval from a Southampton University Ethics Committee was also required.

The RUTI trial is the first trial in the UK of a Chinese herbal remedy registered as an Investigational Medicinal Product. Obtaining ethical and regulatory approval has been a challenging educational and research experience for us as researchers and the various regulatory authorities with who we have worked closely in co-operation. Our first major outcome is that we have managed to negotiate the complex process successfully. This means that we have established a precedent to enable subsequent trials of CHM to take place. The RUTI trial is now recruiting, and we will be able to provide data on the feasibility and safety of using and researching CHM within primary care. This trial will also provide a comparison of individualised versus standardised herbal treatment that will help inform the design of future trials in herbal medicine. Finally, the RUTI trial will provide provisional estimates of the variance of change in continuous outcomes which, if warranted, could be used to inform a power calculation for a larger, more definitive trial for CHM as a treatment for RUTIs.

### Trial status

Recruiting is ongoing.

## Abbreviations

CAM, complementary or alternative medicine; CHM, Chinese herbal medicine; CRN, clinical research network; GP, general practitioner; RCHM, Register of Chinese Herbal Medicine; RUTIs, recurrent urinary tract infections; UTIs, urinary tract infections

## Additional files

Additional file 1: SPIRIT Checklist. SPIRIT-Checklist-download-8Jan13.doc. Recommended items to address in a clinical trial protocol and related documents. (DOC 121 kb) Additional file 2: Consent form. (DOCX 116 kb) Additional file 3: Participant information sheet. (DOC 58 kb) Additional file 4: Summary of product characteristics. (PDF 140 kb)
