# Supplementary material for: Standardised Chinese herbal treatment delivered by GPs compared with individualised treatment administered by practitioners of Chinese herbal medicine for women with recurrent urinary tract infections (RUTI): study protocol for a randomised controlled trial
Source: Trials. 2016 Jul 27;17:358. doi: 10.1186/s13063-016-1471-5 (PMC4962349; doi:10.1186/s13063-016-1471-5)
Supplement: Additional file 2: — Consent form. (DOCX 116 kb) [file 13063_2016_1471_MOESM2_ESM.docx]

**RUTI Trial CONSENT FORM**

Centre Number:

Study Number: **EudraCT** 2013-004657-24

Participant Identification Number for this trial:

**CONSENT FORM**

**Study title**: **A double blind, randomised controlled, feasibility study exploring the role of Chinese herbal medicine in the treatment of recurrent urinary tract infections.**

Name of Researcher:

Please initial box

1. I confirm that I have read the information sheet dated.................... (version............) for the above study. I have had the opportunity to consider the information, ask questions and have had these answered satisfactorily.
2. I understand that my participation is voluntary and that I am free to withdraw at any time
   without giving any reason, without my medical care or legal rights being affected.
3. I understand that relevant sections of my medical notes and data collected during the study, may be looked at by individuals from the University of Southampton, from regulatory authorities or from the NHS Trust, where it is relevant to my taking part in this research. I give permission for these individuals to have access to my records.
4. I understand that the information collected about me may be used to support other research in the future organised by The University of Southampton, and may be shared anonymously with other researchers.
5. I agree to my General Practitioner being informed of my participation in the study.
6. I am happy to be contacted regarding other unspecified research projects. I therefore consent to the University retaining my personal details on a database, kept separately from the research data detailed above. The ‘validity’ of my consent is conditional upon the University complying with the Data Protection Act and I understand that I can request my details be removed from this database at any time.
7. I agree to take part in the above study.

***Data Protection***

*I understand that information collected about me during my participation in this study will be stored on a password-protected computer. All files containing any personal data will be made anonymous.*

Name of Participant Date Signature

Name of Person Date Signature

taking consent
