# Supplementary material for: Standardised Chinese herbal treatment delivered by GPs compared with individualised treatment administered by practitioners of Chinese herbal medicine for women with recurrent urinary tract infections (RUTI): study protocol for a randomised controlled trial
Source: Trials. 2016 Jul 27;17:358. doi: 10.1186/s13063-016-1471-5 (PMC4962349; doi:10.1186/s13063-016-1471-5)
Supplement: Additional file 3: — Participant information sheet. (DOC 58 kb) [file 13063_2016_1471_MOESM3_ESM.doc]

Participant Information Sheet

**Study Title**: **A double blind, randomised controlled, feasibility study exploring the role of Chinese herbal medicine in the treatment of recurrent urinary tract infections.**

**Researcher**: Andrew Flower PhD **Ethics number**: 7985 **EudraCT** 2013-004657-24

**Please read this information carefully before deciding to take part in this research. If you are happy to participate you will be asked to sign a consent form.**

**What is the research about?**

This research is part of a National Institute of Health Research (NIHR) post-doctoral fellowship awarded to Andrew Flower PhD to investigate the possible contribution that Chinese herbal medicine (CHM) could make to the treatment of recurrent urinary tract infections (RUTIs). Currently CHM has not been subjected to the same rigorous testing, review, and oversight as conventional medicine and is not currently commissioned by the NHS. We are going to explore whether CHM helps to prevent RUTIs whether it has any benefits in improving quality of life and if there are any side effects from CHM treatment. We are also going to evaluate how people with RUTIs feel about taking CHM and whether it is a form of treatment that could administered by your GP or whether it needs to be administered by a trained CHM practitioner.

This research is being paid for by the NIHR and the University of Southampton is acting as the legal sponsor for the trial.

**Why have I been chosen?**

Your GP has clarified you as being aged between 18 and 65 and have had 3 or more recurrent urinary tract infections in the previous 12 months, with at least one laboratory confirmation that it is a bacterial infection, then you may be eligible to take part in the trial. We have written to you to ask if this is something you would like to consider.

**Do I have to take part?**

No. It is entirely up to you if you want to take part in this research. The study team will discuss the treatment options available to you. If you chose not to take part then it will not in any way affect the treatment you currently have from your GP.

**What will happen to me if I take part?**

If you would like to take part then you will receive a telephone call to check that you are eligible to take part in the trial. There may be reasons, for example if you get regular kidney infections, or if you have just started a new type of antibiotic or a new course of antibiotic prophylaxis, that we won’t be able to include you in this trial.

If you can be included then we will ask you to attend a meeting with the practice nurse at your local GP practice. You will then be asked to read through and sign a consent form saying you understand what the trial involves and that you are willing to take part. You will be provided with the key questionnaires and forms which we would like you to use during the trial. These will include the symptom diaries to be completed if you get an infection and monthly diaries that we will ask you to use for the duration of the trial to record details about your urination and any signs of infection. At the end of the trial we will ask you to complete three questionnaires that will provide us with an overall summary of your experience during the trial.

The nurse will also ask for your consent to take a sample of your blood to check your liver and kidney function. This sample of blood (approximately 1 teaspoon) will be immediately processed and will not be stored for future analysis during this trial.

You will then be provided with 2 herbal remedies. One will be used in the event of an acute infection. The second will be used when there is no infection and will be used as a herbal prophylaxis. The herbs will be provided as 400mg cellulose capsules in sealed, plastic containers. You will be asked to take 4 pills twice a day when using the preventative formula and 4 pills 4 times a day in the event of an acute infection. Pills should be taken half an hour before food or on an empty stomach with warm water. During the trial, if you are concerned about your symptoms, you are free to consult with your GP and, if necessary, use conventional medicines such as antibiotics for any acute infections.

As this trial is a placebo-controlled trial half of the participants will be randomized to a group taking a placebo herbal remedy. This means that it will not contain any active herbal treatment. Neither the participant nor the person who gives you the herbs will know which group you are in. Only when the trial has been completed will we know who has been allocated to which group. We will write to tell you once the trial has concluded. For reasons that are not currently well understood, these inactive remedies can still result in symptomatic improvements and even side effects.

You will be asked to attend a second consultation at 4 weeks to collect more herbs, check that outcomes are being completed correctly, and to receive another 12 weeks’ of herbal supplies. The practice nurse will also take another blood sample to check your liver and kidney function and to make sure you are not having any side effects from the herbs.

You will then be asked to attend a final interview at week 16 of the trial where your questionnaires will be collected and a final sample of blood will be taken. In the intervening period we will phone you once a month to check that all is well and that outcomes are being recorded.

We will then ask you to continue keeping a record of any urinary infections that you experience for 6 months after you stop taking the herbs.

Some women will be asked if they would be willing to be interviewed about their experiences of taking part in the trial. This will take place after the trial has been completed and will involve an hour interview to be held either at your GP practice or at your home-depending on what is more convenient for you. These interviews will be recorded so we can analyse the data on them. Only research team members and a professional transcriber will have access to the recordings and they will be deleted after the study has been published. Anonymised quotes will be included in research reports only with the permission of the interviewee.

**Are there any benefits in my taking part?**

You may find the treatment reduces the frequency and severity of your recurrent urinary tract infections. However these benefits have not been proven and it is the aim of this research to explore whether CHM can help in these circumstances. If this trial suggests that CHM may be a useful treatment then it will support the need for a larger future trial that should be able to provide more definitive evidence. If this does prove to be the case then these herbal products could become more widely available to other women with the same condition.

**Are there any risks involved?**

In CHM it is known that the herbs can cause some digestive upset like transient nausea or loose bowels. However these usually only last for 2-3 days and generally the herbs are well tolerated. In very rare instances the herbs can cause abnormal liver or kidney function. You will be given blood tests at the beginning of the trial, after 4 weeks of taking the herbs and again at the end of the trial to measure your liver and kidney function and to ensure that you can tolerate the Chinese herbs. There may also be unknown side effects from CHM and during the course of the trial, if you develop any unusual symptoms or have any concerns about the Chinese medicine you will be able to phone or e mail the Principal Investigator of the trial who will be able to advise you on the appropriate course of action to take. You will also be able to visit your GP if you develop problems during the trial.

Because we are not certain whether Chinese herbs can be taken during pregnancy we ask all participants to try to avoid conceiving whilst on the trial and to inform us in the event that that do become pregnant. If you are already pregnant you will not be able to participate in the trial.

The blood tests will be administered by a qualified practice nurse. Some people may feel faint when giving a blood sample. If this occurs the practice nurse will advise you what to do to help you recover from these feelings.

Lactose is used as a component of the herbal medicine capsules so if you are lactose intolerant you should not participate in the trial.

There is some evidence that one of the herbs we will be using in the trial may decrease levels of some conventional drugs. For these reasons if you are already taking immune-suppressant medication, medications for cardio-vascular disease (including warfarin and digoxin), lithium, ongoing anti-viral treatment, or having treatment for cancer you will be unable to take part in the trial. If you have any concerns about any medication you are currently taking please mention these to the member of the research time who will contact you if you wish to proceed with the trial.

**Will my participation be confidential?**

Any data from this research will be stored according to the University Data Protection policy. Written and digitally recorded data will be stored on a password-protected computer that will be kept in a secure environment. Interviews will be coded and real names will be deleted to preserve confidentiality. Any emails sent by research participants will have their content coded and preserved within a password protected Word document but contact details and original emails will be deleted. Any papers connected with this trial will be kept in secure University storage for10 years and will only be viewed by the research team.

All participants who take part in the trial will be given a code and that code, rather than your name, will be used to identify you in the analysis and write up of the trial. Your GP practice will have details of your involvement in the trial so we cannot guarantee complete anonymity but we can assure you that any researcher will only be aware of your code and not your personal details. Anything you report to the practice nurse or researcher during the trial will also be treated as confidential.

**What happens if I change my mind?**

If, at any time, you decide that you no longer wish to proceed with this research you have the right to withdraw from the trial. This will not in any way affect your legal rights to access treatment or your access to routine care for your condition.

**What happens if something goes wrong?**

In the unlikely case of concern or complaint regarding this research project you can contact the Research Governance Manager on 02380 595058, or by email at [rgoinfo@soton.ac.uk](mailto:rgoinfo@soton.ac.uk)

**Where can I get more information?**

If you require any more information or have any concerns regarding the research that you wish to discuss you can contact Andrew Flower (02380 241073), [Andrew.Flower@soton.ac](mailto:flower.power@which.net)
