# Supplementary material for: Standardised Chinese herbal treatment delivered by GPs compared with individualised treatment administered by practitioners of Chinese herbal medicine for women with recurrent urinary tract infections (RUTI): study protocol for a randomised controlled trial
Source: Trials. 2016 Jul 27;17:358. doi: 10.1186/s13063-016-1471-5 (PMC4962349; doi:10.1186/s13063-016-1471-5)
Supplement: Additional file 4: — Summary of product characteristics. (PDF 140 kb) [file 13063_2016_1471_MOESM4_ESM.pdf]

APPENDIX 5

Table 2: RUTI Trial Preventative Formula (RUTI-p)

**Name (Botanical, PinYin, Common names)**<br><br>**Traditional daily dose (grams)**	**Active compounds**	**Pharmacological effects**	**Clinical trial data**	**Adverse effects/Toxicity**	**Traditional Use**
Astragalus membranaceus Moench<br><br>Huang Qi<br><br>Milkvetch root<br><br>15-30g	The roots of Astragalus membranaceus contain biologically active saponins (including 11 astragalosides and one soyasaponin), flavones including kaempferol, quercetin); and polysaccharides (including Astragalans I,II and III.[1,2,3]	Supports immune function by eg a) increasing phagocytic activity of macrophages, (b) increasing proinflammatory cytokines IL1, IL6 and TNF, and (c) increasing levels of lymphocyte stimulatory IL2 and IL2 receptor expression[1,2,3,4.].<br>May enhance TLR4 gene expression in bladder epithelial cells to promote local neutrophil and cytokine anti microbial activity[5].<br><br>May enhance physiological metabolism of renal cells (eg increasing glycogen granules, acid	Preliminary data supporting its use for asthma, chemotherapy induced leucopenia and viral myocarditis[6,7].<br><br>Demonstrated immunomodulatory and anti microbial effects in a number of common infections[8,9,10] including UTIs[11].	No adverse reactions observed in mice with dosages as high as 100g/kg of the herb.<br><br>LD_{50} of the decoction of the herb administered by intraperitoneal injection was 39.82g/kg.[1]	Invigorates the vital energy, protects against infection, promotes healing and regulates the 'water pathways'[2,3].

		phosphatase and succinic dehydrogenase).[1]<br><br>Mild diuretic effect (0.2g/kg increased urine output by 64%)[1]<br><br>Anti-inflammatory effect (may reduce histamine induced increase in vascular permeability)[1]<br><br>Hepatoprotective effect [2]			
Sophora flavescens Aiton<br><br>Ku Shen<br><br>6-15g	The known chemical components of Sophora flavescens include alkaloids (3.3%) - principally matrine and oxymatrine; flavonoids (1.5%), alkylxanthones, quinones, triterpene glycosides, fatty acids, and essential oils[1,3.].	In vitro research shows the flavonoid extracted from Ku Shen (Kurarinone) has a strong inhibitory action on the growth of MRSA (Methicillin-Resistant *Staphylococcus aureus*) and VRE (Vancomycin resistant Enterococci) bacteria[12].<br><br>Sophora flavescens also has an inhibitory in vitro action against E Coli (Zhu), and B-haemolytic Streptococcus[1].	There is preliminary evidence supporting the use of Sophora flavescens for asthma, psoriasis, eczema, arrhythmia, and to prevent leukopenia during cancer chemotherapy[2].	Occasional reports of mild, transient, gastrointestinal disturbances[2].<br><br>The LD_{50} for injection of the herbal extract in mice corresponded to a dose of 15 grams of herb per kg (in human terms, this corresponds, roughly, to a single dose of 1 kg of the herb)[1].	Clears Heat and drains Damp to treat diarrhea, skin diseases, and relieve painful, frequent urination[3].

Lindera aggregata var. aggregate<br><br>Wu Yao<br><br>Lindera Root<br><br>6-12g	Contains a number of alkaloids, volatile oils and sesquiterpene esters[1,3].	In vitro research suggests this herb exhibits inhibitory effects on the production of inflammatory mediators from macrophages via blocking NF-kappaB and MAPKs signaling pathways[13].	In vitro analysis suggests Lindera essential oils have an anti-bacterial action against common bacteria including E Coli[14].<br><br>Preliminary evidence that this herb might help slow down diabetic nephropathy[15].	No reported adverse events.	Circulates vital energy and alleviates pain, warms the 'kidney' and regulates urination[2,3.].

[1] Zhu YP (1998). Chinese Materia Medica: Chemistry, Pharmacology & Applications. Harwood Academic Publications.

[2] Chen JK & Chen TT (2004). Chinese Medical Herbology and Pharmacology. Art of Medicine Press.

[3] Bensky D, Clavey S, Stoger E (2004). Chinese Herbal Materia Medica (3rd Edition). Eastland Press.

[4] Cho WC, Leung KN. In vitro and in vivo immunomodulating and immunorestorative effects of Astragalus membranaceus. J Ethnopharmacol. 2007;113:132–141.

[5] Spelman K, Burns J, Nichols D, Winters N, Ottersberg S, et al. Modulation of cytokine expression by traditional medicines: a review of herbal immunomodulators. Altern Med Rev. 2006;11:128–150.

[6] Zhang C, Zhu C, Ling Y, Zhou X, Dong C, Luo J, Liu Y. The clinical value of huangqi injection in the treatment of leucopenia: a meta-analysis of clinical controlled trials.PLoS One. 2013 Dec 12;8(12).

[7] Liu JP, Yang M, Du XM. Herbal medicines for viral myocarditis. Cochrane Database Syst Rev. 2004;(3):CD003711

[8] Yesilada E, Bedir E, Caliş I, Takaishi Y, Ohmoto Y. Effects of triterpene saponins from Astragalus species on in vitro cytokine release. J Ethnopharmacol. 2005;96:71–77

[9] Shon YH, Kim HJ, Nam KS. Effect of Astragali radix extract on lipopolysaccharide-induced inflammation in human amnion. Biol Pharm Bull. 2002;25:77–80.

[10] Tan BK, Vanitha J. Immunomodulatory and antimicrobial effects of some traditional chinese medicinal herbs: a review. Curr Med Chem. 2004;11:1423–1430
[11] Xiaolin Yin, Lei Chen, Ying Liu, Jianling Yang, Cuiqing Ma, Zhiyan Yao, Lijuan Yang, Lin Wei, Mingyuan Li, Enhancement of the innate immune response of bladder epithelial cells by Astragalus polysaccharides through upregulation of TLR4 expression, Biochemical and Biophysical Research Communications, Volume 397, Issue 2, 25 June 2010, Pages 232-238
[12] Chen L, Cheng XF, Shi WY et al Letter to the editor: Inhibition of Growth of Streptococcus mutans, Methicillin-Resistant Staphylococcus aureus, and Vancomycin-Resistant Enterococci by Kurarinone, a Bioactive Flavonoid Isolated from Sophora flavescens. J Clin Microbiology. 2005;7:3574–3575
[13] Luo Y, Liu M, Yao X, Xia Y, Dai Y, Chou G, Wang Z. Total alkaloids from Radix Linderae prevent the production of inflammatory mediators in lipopolysaccharide-stimulated RAW 264.7 cells by suppressing NF-kappaB and MAPKs activation. Cytokine. 2009; 46:104-10. Epub 2009 Feb 26.
[14] Yan RW, Yang Y, Zeng YY, Zou GL. Cytotoxicity and antibacterial activity of Lindera strychnifolia essential oils and extracts.Journal of Ethnopharmacology 2009;30:451–455.
[15] Ohno, T., Takemura, G., Murata, I., Kagawa, T., Akao, S., Minatoguchi, S., Fujiwara,T., Fujiwara, H. Water extract of the root of Lindera strychnifolia slows down the progression of diabetic nephropathy in db/db mice. Life Sciences. 2005b; 77:1391–1403.

APPENDIX 6 RUTI Trial Acute formulae (RUTI-a)

Name (Botanical, PinYin, Common names)<br><br>Traditional daily dose (grams)	Active compounds	Pharmacological effects	Clinical trial data	Adverse effects/Toxicity	Traditional Use
Oldenlandia diffusa (Willd.) Roxb.<br><br>Bai Hua She She Cao<br><br>Oldenlandia<br><br>15-60g	Contains Iridoid and Flavonoid glycosides (eg Oldenlandosides A and B), Anthroquinones, Triterpenes, and p-coumaric acid[1,2,3].	Has demonstrated an in vitro anti-bacterial action against Staphylococcus aureus. E coli, Pseudomonas aeruginosa[4].<br><br>Has anti-inflammatory effect by inhibiting the production of tumor necrosis factor (TNF)-α, interleukin (IL)-6 and prostaglandin E(2) (PGE(2)[5].<br><br>May increase leukocyte phagocytic activity[1].<br><br>Oldenlandia diffusa extract effectively inhibited the growth of several cancer cell lines[6].	Commonly used in Chinese clinical trials as part of a formula for the treatment of acute urinary tract infections.<br><br>Also used in many trials for the treatment of hepatitis and a number of different cancers.[1,2,3]	LD_{50} of the extract of the herb by intraperitoneal administration was 104g/kg[2] [1]	Clears Heat and eliminates toxins used for skin diseases, for snakebites, urinary tract infections and recently as an anti-cancer herb.[2,3.]

Phellodendron amurense Rupr.<br><br>Huang Bai<br><br>Amur Cork tree<br><br>9-15g	Contains a number of isoquinoline alkaloids (bereberine is the best documented and is present at levels ranging from 0.6-2.5%). Triterpenes, phytosterols, and phenolic compounds have also been isolated.[1,3.]	Anti-inflammatory effects and anti-microbial effects noted in vitro and in vivo-including against MRSA when used singly or in conjunction with ampicillin and oxacillin.[7,8,9]	Preliminary evidence of the effectiveness of the herbs in treating acute and chronic bacillary dysentery[1].	No side effects reported for oral administration.<br><br>LD_{50} after intraperitoneal administration was 2.7g/kg[1].	Clears Heat, dries Damp and clears toxins traditionally used for infectious diarrhoea, urinary tract infections, and skin diseases[2,3].
Desmodium styracifolium (Osbeck) Merr.<br><br>Jin Qian Cao<br><br>Gold Coin herb<br><br>15-30g	Contains a number of flavonoids<br>and alkaloids, followed by terpenoids, steroids, phenols, phenylpropanoids, glycosides and a number of volatile oils [1,2,3].	Anti-inflammatory action[10]<br><br>Prevents and treats renal stones [1,2,10]	Commonly used in clinical trials for the treatment of UTIs [1,2,3,10,11].	No reports of adverse reactions despite large dosages administered[1].	Clears Heat, drains Damp and dissolves stones for the treatment of urinary tract infections, kidney, bladder and gall bladder stones. Also used for jaundice, skin diseases[2,3].

[1] Zhu YP (1998). Chinese Materia Medica: Chemistry, Pharmacology & Applications. Harwood Academic Publications.

[2] Chen JK & Chen TT (2004). Chinese Medical Herbology and Pharmacology. Art of Medicine Press.

[3] Bensky D, Clavey S, Stoger E (2004). Chinese Herbal Materia Medica (3rd Edition). Eastland Press.

[4] Bian CM. Study on the Anti-microbial Effect of the Extraction Oldenlandia diffusa (Willd) Roxb [J]. LiShiZhen Medicine and Materia Medica Research: 2010:10: 991-2.

[5] Kim SJ, Chung WS, Kim SS, Ko SG, Um JY. Antiinflammatory effect of Oldenlandia diffusa and its constituent, hentriacontane, through suppression of caspase-1 activation in mouse peritoneal macrophages. Phytother Res. 2011;10:1537-46.

[6] Gupta S, Zhang D, Yi J, Shao J. Anti-cancer activities of Oldenlandia diffusa. J Herb Pharmacother. 2004;4:21-33

[7] Chen ML, Xian YF, Ip SP, Tsai SH, Yang JY, Che CT. Chemical and biological differentiation of Cortex Phellodendri Chinensis and Cortex Phellodendri Amurensis. Planta Med. 2010 Oct;76:1530-5.

[8] Park EK, Rhee HI, Jung HS, Ju SM, Lee YA, Lee SH, Hong SJ, Yang HI, Yoo MC, Kim KS.
Antiinflammatory effects of a combined herbal preparation (RAH13) of Phellodendron amurense and Coptis chinensis in animal models of inflammation. Phytother Res. 2007;21(8):746-50.

[9] Yu HH, Kim KJ, Cha JD, Kim HK, Lee YE, Choi NY, You YO. Antimicrobial activity of berberine alone and in combination with ampicillin or oxacillin against methicillin-resistant Staphylococcus aureus. J Med Food. 2005;8(4):454-61.

[10] Ma XQ, Zheng CJ, HU CL, Rahman K, Qin LP The genus Desmodium (Fabaceae)-traditional uses in Chinese medicine, phytochemistry and pharmacology Journal of Ethnopharmacology 2011;138:314– 332.

[11] Peng B, Liu Q, Hou J, Zhou SC. Lysimachia Christinae Hance Tablet in treatment of urinary tract infection:a randomized,multicenter,controlled clinical study. Modern Medicine & Health 2011-11.
